# Prognostic Value of Left Ventricular Function and Mechanics in Pulmonary Hypertension: A Pilot Cardiovascular Magnetic Resonance Feature Tracking Study

**DOI:** 10.3390/medicina55030073

**Published:** 2019-03-20

**Authors:** Lina Padervinskienė, Aušra Krivickienė, Deimantė Hoppenot, Skaidrius Miliauskas, Algidas Basevičius, Irena Nedzelskienė, Antanas Jankauskas, Paulius Šimkus, Eglė Ereminienė

**Affiliations:** 1 Department of Radiology, Medical Academy, Lithuanian University of Health Sciences, LT 44307 Kaunas, Lithuania; algidas.basevicius@kaunoklinikos.lt (A.B.); jankauskas.antanas@gmail.com (A.J.); pauliusimkus@gmail.com (P.Š.); 2 Department of Cardiology, Medical Academy, Lithuanian University of Health Sciences, LT 44307 Kaunas, Lithuania; krivickiene.ausra@gmail.com (A.K.); eglerem@yahoo.com (E.E.); 3 Department of Pulmonology, Medical Academy, Lithuanian University of Health Sciences, LT 44307 Kaunas, Lithuania; deimante.hoppenot@lsmuni.lt (D.H.); skaidrius.miliauskas@kaunoklinikos.lt (S.M.); 4 Department of Dental and Oral Diseases, Medical Academy, Lithuanian University of Health Sciences, LT 44307 Kaunas, Lithuania; irena.nedzelskiene@lsmuni.lt

**Keywords:** pulmonary hypertension, left ventricular, feature tracking, cardiovascular magnetic resonance

## Abstract

*Background and objective*: Cardiovascular magnetic resonance (CMR) - based feature tracking (FT) can detect left ventricular (LV) strain abnormalities in pulmonary hypertension (PH) patients, but little is known about the prognostic value of LV function and mechanics in PH patients. The aim of this study was to evaluate LV systolic function by conventional CMR and LV global strains by CMR-based FT analysis in precapillary PH patients, thereby defining the prognostic value of LV function and mechanics. *Methods*: We prospectively enrolled 43 patients with precapillary PH (mean pulmonary artery pressure (mPAP) 55.91 ± 15.87 mmHg, pulmonary arterial wedge pressure (PAWP) ≤15 mmHg) referred to CMR for PH evaluation. Using FT software, the LV global longitudinal strain (GLS) and global circumferential strain (GCS), also right ventricular (RV) GLS were analyzed. *Results*: Patients were classified into two groups according to survival (survival/non-survival). LV GLS was significantly reduced in the non-survival group (−12.4% [−19.0–(−7.8)] vs. −18.4% [−22.5–(−15.5)], *p* = 0.009). By ROC curve analysis, LV GLS > −14.2% (CI: 3.229 to 37.301, *p* < 0.001) was found to be robust predictor of mortality in PH patients. Univariable analysis using the Cox model showed that severely reduced LV GLS > −14.2%, with good sensitivity (77.8%) and high specificity (93.5%) indicated an increase of the risk of death by 11-fold. LV GLS significantly correlated in PH patients with RV ESVI (*r* = 0.322, *p* = 0.035), RV EF (*r* = 0.444, *p* < 0.003). *Conclusions*: LV systolic function and LV global longitudinal strain measurements using CMR-FT correlates with RV dysfunction and is associated with poor clinical outcomes in precapillary PH patients.

## 1. Introduction

Pulmonary hypertension (PH) is defined as an increase in mean pulmonary artery pressure (mPAP) >25 mmHg evaluated by right heart catheterisation (RHC) [1]. Over time, increased pulmonary artery pressure eventually causes vascular remodeling and right ventricular (RV) dysfunction. 

The RV and left ventricular (LV) interact, sharing a common pericardial sac and interventricular septum. Frequently, RV dysfunction reflects clinical PH worsening. The prognostic value of RV mass, volume and function in PAH was shown in several studies [2,3,4,5]. Moreover, there are some data supporting the theory that RV derangement also may influence LV function and mechanics [6,7]. Previous studies showed that LV diastolic filling and systolic function may be affected by direct interventricular septum interaction [6,7,8,9,10]. This leads not only to RV impairment, but also to LV dysfunction [11]. 

LV structure and functional impairment may be evaluated using non-invasive technologies, such as echocardiography or cardiovascular magnetic resonance (CMR). In the last few decades, additional technologies have appeared, so the evaluation of the heart has expanded from being structural/functional to mechanical in nature. Echocardiographic speckle tracking, the first modality used for tissue tracking in cardiac imaging [12], revealed that not only volumetric, but also mechanical evaluation of the ventricles can be performed in patients with cardiovascular and pulmonary pathology [13,14,15,16,17,18], and may be useful in taking different clinical decisions. Since echocardiography images have a lower signal-to-noise ratio than CMR and are highly dependent on the image quality (operator dependence, patient body habitus (echo window)) [19], feature tracking (FT) has become a more attractive technology for the evaluation of myocardial deformation. Myocardial strain has been shown to be more sensitive than EF in detecting subclinical ventricular contractile dysfunction [20]. 

The relatively new FT technique has been used in different pathologies to evaluate ventricular mechanics, and its prognostic value has been assessed [21,22,23]. FT can detect LV strain abnormalities in PH patients [15], but little is known about prognostic value of LV function and mechanics metrics in PH patients. Therefore, the aim of this study was to evaluate the LV systolic function by conventional CMR and LV global strains by FT analysis in PH patients and to define the prognostic value of these parameters.

## 2. Materials and Methods

### 2.1. Patients Selection and Testing

This study consisted of 55 prospectively included patients with confirmed precapillary PH on right heart catheterisation (mean pulmonary artery pressure (mPAP) 55.91 ± 15.87 mmHg, pulmonary arterial wedge pressure ≤15 mmHg) from November 2012 to October 2017 in the Hospital of Lithuanian University of Health Sciences Kauno klinikos. The study protocol conforms to the ethical guidelines of the 1975 Declaration of Helsinki as reflected in a prior approval by the Regional Biomedical Research Ethics Committee of the Lithuanian University of Health Sciences (ID No. BE-2-23, approved on 5th of June 2015). Also, the study was registered in Protocol Registration and Results System (ClinicalTrials.gov ID no. NCT03377673). Informed consent was obtained from each patient. At the time of evaluation, all patients were receiving standard precapillary PH therapy. All patients underwent CMR imaging scans. Twelve patients were excluded from the analysis for the following reasons: documented coronary and valvular heart disease (3 patients), underlying cardiomyopathy (1 patient), shortness of breath (3 patients), low-quality studies due to arrhythmia (3 patients), claustrophobia (2 patients). During the study, referring physicians were informed about CMR findings, so these findings influenced clinical decisions. The mean interval between CMR and RHC was 15.6 ± 12.7 days. FT analysis of LV and RV strains was performed. 

The following clinical characteristics of the patients were analyzed at the time of initial evaluation: age, sex, body surface area (calculated from height and weight), New York Heart Association functional classification (NYHA), six minutes walking test (6MWT), mPAP and B-type natriuretic peptide (NT-pro BNP) level. 

### 2.2. CMR Measurements

CMR was performed using a 1.5T whole-body system (Siemens Aera, Siemens Medical Solutions; Erlangen, Germany). End-diastole and end-systole of the ventricles were defined as maximum and minimum volumes on balanced steady-state free precession (bSSFP) sequences. End-diastolic volume (EDV), end-systolic volume (ESV) and ejection fraction (EF) of the ventricles were calculated in standard cine images using MR analysis software system (syngo.via; Siemens Healthcare). Indices of the ventricle’s volumes (end-diastolic volume index (EDVI) and end-systolic volume index (ESVI)) were calculated based on body surface area. Also, the right ventricular mass index (RVMI) was measured and late gadolinium enhancement (LGE) assessed.

### 2.3. FT Analysis

**Figure 1:**
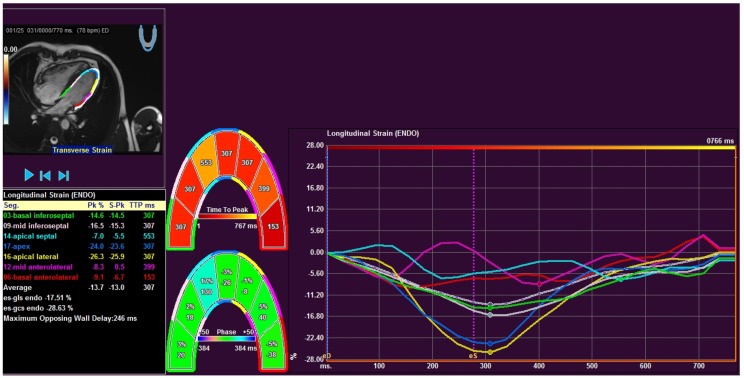
Measurement window of feature tracking software of patient with precapillary PH from four chamber long axis view. Middle figures show graphical display of 7 segments: three septal, three lateral and apical segments. Right panel depicts the corresponding longitudinal strain values. Left black box shows peak values and time to peak for corresponding segments.

CMR images were analysed by two experienced radiologists using a commercial FT software package (Medis Suite QStrain 2.0; Medis Medical Imaging Systems bv, Leiden, The Netherlands). Endocardial borders of the ventricles in end-diastole and end-systole were marked semi-automatically throughout the cardiac cycle on standard CMR balanced steady-state free precession (bSSFP) sequences. The contours were checked and manually adjusted if needed. LV global longitudinal strain (GLS) was calculated by averaging the strain curves of two-chamber, three-chamber and four-chamber long-axis views (Figure 1). LV global circumferential strain (GCS) was calculated by averaging the strain curves of basal, mid and apical segments obtained from the short-axis views. RV regional (septal and free wall) longitudinal strains and GLS was calculated in the cardiac four-chamber long-axis view. 

### 2.4. Data Analysis

Statistical analyses were performed using SPSS 22.0 package (SPSS, Chicago, IL, USA). Continuous variables were expressed as mean ± standard deviation (SD), skewed variables as median (interquartile range). Intra- and interobserver variabilities for strain values were assessed by repeated analysis of 10 randomly selected patients. A Chi square (χ^2^) test was used for qualitative parameters. For continuous variables, differences between two groups were compared using non-parametric Mann-Whitney U test. The relationship between variables was assessed using the Spearman correlation coefficient. In order to assess minimally false negative and minimally false positive results with greatest accuracy, the method of ROC (Receiver Operating Characteristics) curve was used. Survival curves were established by the Kaplan-Meier estimation method. Cox regression analysis was used to identify independent predictor of outcomes. Two-tailed probability values at *p* < 0.05 were considered statistically significant.

## 3. Results

Forty-three PH patients were enrolled in the analysis during the period between November 2012 to October 2017. Patients were divided into two groups according to the survival (survival/non-survival). During the mean follow-up period (30.7 ± 19.9 months) 12 patients died, due to: cardiovascular pathology (5 patients), arrhythmia (2 patients), infections (5 patients) (primary endpoint of the study—non-survival group). Distribution of age, gender, etiology and NYHA functional class, mean pulmonary artery pressure and six-minute walk test did not differ between survival and non-survival patient group (Table 1). B-type natriuretic peptide level was significantly higher in non-survival group. All PH patients were undergoing treatment with the specific therapy available in our country for PAH (i.e., endothelin antagonist, PDE 5 inhibitors, prostacyclin analogues) at the time of the study (69% monotherapy, 31% combination therapy). The specific therapy did not differ between survival and non-survival groups (*p* = 0.328).

Patient CMR and FT measurements data are summarised in Table 2. CMR FT parameters demonstrated low intra- and inter-observer variability indicated by the intraclass correlation coefficient: 0.97/0.86 for RV-GLS, 0.96/0.95; 0.98/0.92 for LV-GLS and LV-GCS, respectively (*p* < 0.05).

RVEF had a tendency to be lower (37.5% [18.8–41.8] vs. 42.0% [32.0–47.0], *p* = 0.088) and the indices of RVESVI and RVEDVI had a tendency to be higher (63.5 mL/m^2^ [45.8–91.3] vs. 48.0 mL/m^2^ [37.0–60.0], *p* = 0.06 and 87.0 mL/m^2^ [76.0–112.25] vs. 82.0 mL/m^2^ [68.0–101.0], *p* = 0.24, respectively) in non-survival patients group, but these differences did not reach statistical significance. RVMI did not differ between survival and non-survival patient groups. Specific LGE pattern (ventricles insertion points with/without spread into interventricular septum) was found in both PH patient groups. The frequency of LGE did not differ between the survival and non-survival groups.

Indices of left ventricular volumes were within normal limits in all study PH patients. However, LVEF was significantly lower in non-survival group.

The LV GLS was significantly reduced in non-survival PH patients (−12.4% [−19.0–(−7.8)] vs. −18.4% [−22.5–(−15.5)], *p* = 0.009). Although the LV GCS was reduced in non-survival group, the difference was not statistically significant (−28.5% [−34.2–(−23.5)] vs. −32.0% [−36.8 –(−28.2)], *p* = 0.22). The measurements of RV GLS did not differ between groups.

An increase of distance, measured during Cox regression analysis test, by 1, reduces LV GLS, increases RVESVI and decreases RV and LV EF Hazard Ratio (HR) for non-survival.

**Figure 2:**
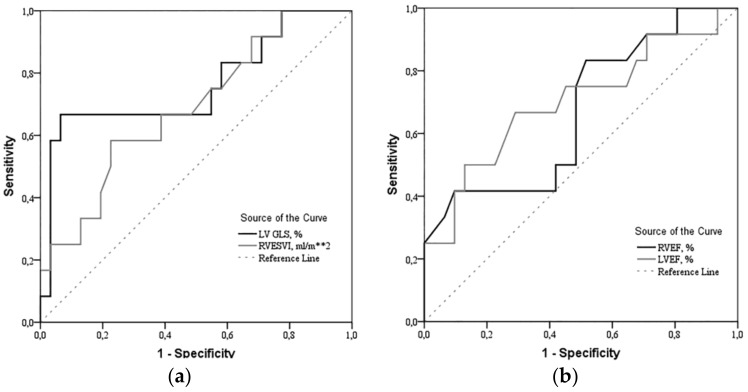
Receiver Operating Characteristics (ROC) curves of (**a**) LV GLS and RV ESVI, (**b**) RVEF and LVEF. LV GLS > −14.2% (CI: 3.229 to 37.301, *p* < 0.001), RVESVI > 60 mL/m^2^ (CI: 0.949 to 9.433, *p* = 0.025), RVEF < 25.5% (CI: 1.109 to 11.053, *p* = 0.016)) and LVEF < 52% (CI: 0.977 to 10.809, *p* = 0.024) were found to be robust predictors of mortality in precapillary pulmonary hypertension patients. LV—left ventricle; GLS—global longitudinal strain; RV—right ventricle; ESVI—end-systolic volume index; EF—ejection fraction.

The results of the ROC curve analysis for the determinants (LV GLS, RVESVI, RVEF and LVEF) are shown in Figure 2.

The LV GLS significantly correlated with poor outcome variables RVESVI (*r* = 0.322, *p* = 0.035), RVEF (*r* = −0.441, *p* = 0.003) in PH patients.

**Figure 3:**
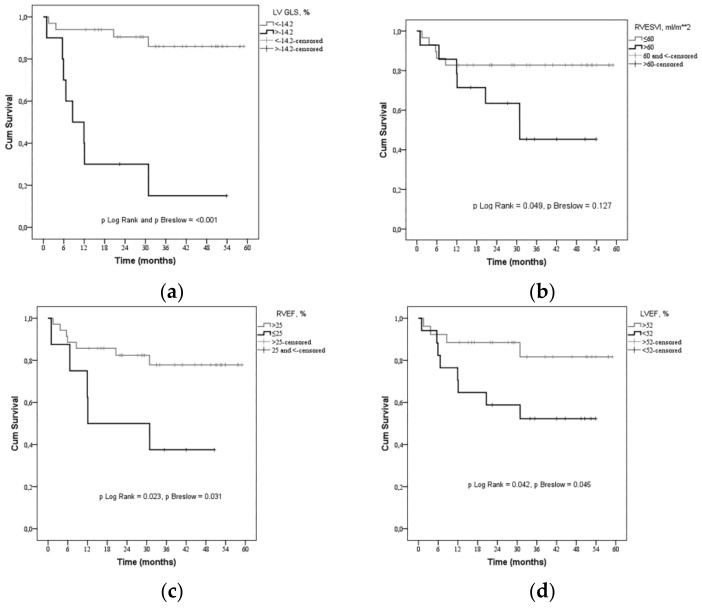
Kaplan-Meier curve—estimation of the time to death by (**a**) LV GLS (>−14.2%), (**b**) RV ESVI (>60 mL/m^2^), (**c**) RV EF (<25.5%) and (**d**) LV EF (<52%). Severely reduced LV GLS > −14.2%, increased RV ESVI > 60 mL/m^2^, decreased RV EF < 25.5% and LV EF < 52% indicate a greater risk of death in precapillary pulmonary hypertension. LV—left ventricle; GLS—global longitudinal strain, RV—right ventricle; ESVI—end-systolic volume index, EF—ejection fraction.

Univariable analysis using the Cox model (Figure 3, Table 3 and Table 4) showed that severely reduced LV GLS > −14.2%, with good sensitivity (66.7%) and high specificity (93.5%) indicated an increase of the risk of death; increased RVESVI > 60 mL/m^2^, despite its limited sensitivity (58.3%), but good specificity (77.4%) tends to increase the risk of death; decreased RVEF < 25.5% with sensitivity 41.7% and good specificity (92.3%) increases the risk of death; LVEF < 52% with good sensitivity 66.7% and specificity (71%) tends to increase the risk of death.

## 4. Discussion

CMR is the gold standard for the evaluation of ventricular volumes and functional parameters because of good inter-study reproducibility [24,25]. It may also provide useful prognostic information at baseline and follow-up [1]. LV functional and mechanical changes in PH, as well as their impact on prognosis, have not been well studied in the past, and robust understanding of the LV mechanical changes in PH is still lacking. Until now, most research has focused on the right ventricular functional, volumetric and mechanical changes in PH. Baggen VJM et al. in their systematic review and meta-analysis concluded that right ventricular ejection fraction, right ventricular end-diastolic and end-systolic volumes indices, and left ventricular end-diastolic volume index were of prognostic importance [26] in PH patients. The results of our study show that the right ventricular end-systolic volume index is significantly increased in a non-survival group compared to a survival group of patients, and this parameter has prognostic importance as well.

The growing progress of tissue tracking technology allows non-invasive evaluation of the parameters of myocardial deformation and provides a better understanding of the mechanical changes of the ventricles [27]. De Siqueira et al. analyzed clinical significance of right ventricular mechanics in PH using FT method by dividing patients into three groups based on RV ejection fraction (> or <50%) and presence of PH disease. They found that right ventricular circumferential strain correlates with disease severity and also showed that right ventricular circumferential and longitudinal strain rates and longitudinal strain adjusted for clinically meaningful covariates are also independently associated with poor outcomes in PH [28]. Yang et al. analyzed 364 patients with different cardiac pathologies and showed that right ventricular global longitudinal strain was significantly associated with major adverse cardiac events. However, patients with pulmonary hypertension were not included in this study [18]. Although our study did not find a statistically significant difference for right ventricular global and regional longitudinal strain parameters between PH groups, there was a trend for regional longitudinal strain (free wall and septum) and global longitudinal strain to be reduced in non-survival group patients. It is possible that the lack of statistical significance was due to PH patient population, because all patients had severely impaired RVEF (<50%). In addition, it should be noted that all the PH patients of this study presented reduced RV strains compared to normal population.

Impairment of left ventricular global circumferential strain has been previously reported in small group of PH patients compared to controls [15]. We demonstrated that the left ventricular global longitudinal strain was significantly reduced in non-survival PH patients compared to patient which survived (mean follow-up period 30.67 ± 17.91 months). Our study is the first to have shown that the reduction of left ventricular global longitudinal strain based on CMR feature tracking is associated with mortality in patients with precapillary PH. These findings suggest that the LV GLS and LV GCS may be related to PH and may have an important relationship to LV mechanics. The reason why changes of LV GLS have not been previously reported [15] may be related to a small sample size and the heterogeneous PH cohort, where patients with mild cardiac dysfunction were included. Our study cohort is also relatively small; therefore, we included only patients with precapillary PH and with a greater impairment of cardiac function (severely increased RV volumes, severely depressed RV systolic function; mildly depressed LV systolic function in non-survival PH group).

Nahum J. et al. analyzed clinical outcomes in heart failure patients using speckle tracking technology and found that severely reduced LV global longitudinal strain >−9% predicted an increase of the risk of cardiac events by 5.1-fold [29]. Buss SJ et al. found that dilated cardiomyopathy patients with reduced LV GLS (>−12.5%), LV GCS (>−13.1%), and LV global radial strain (>15.7%) based on CMR FT analysis had a significantly higher rate of severe cardiac events, and that LV GLS was the most robust predictive factor of cardiac outcomes [22]. Our data demonstrated that parameters of RV impairment (increased ESVI and decreased EF) are associated with reduced LV global longitudinal strain, which was found to be a prognostic factor for PH patient survival. We found that significant reduction of LV GLS > −14.2% and LVEF < 52% are indicators of death risk by 11 and 3.25-fold, respectively. We are in agreement with previous studies regarding the notion that the reduction of the LV GLS is an important predictor of poor outcomes in PH patients. Based on the results of our study, we suggest that PH patient survival might be strongly associated not only with conventional LV functional parameters, but also with the changes with more specific LV mechanical parameters. Since LVEF is mainly dependent on radial myocardial contraction, it is possible that longitudinal contraction, which can be evaluated by FT, might be even more important to clinical outcomes in PH patients.

### Study Limitations

Although our study revealed new scientific data about LV mechanical changes in PH patient outcomes, this study also has some limitations. The heterogeneity of our study population (PH course, active shunts) may limit ability to detect myocardial deformation changes, but since precapillary PH is a rare disease, it is difficult to avoid this limitation. Not all of the initially recruited precapillary PH patients could be included into the analysis, because of documented coronary and valvular heart disease, underlying cardiomyopathy, severe shortness of breath, arrhythmias or claustrophobia. Inclusion of CTEPH patients into analysis (because of different therapeutic approach) might affect the prognostic data. We were unable to analyze RV regional and global CS because of feature tracking software limitations. And finally, the sample size of study subjects could be too small to find more subtle differences between subject groups.

## 5. Conclusions

Increase in right ventricular volumes, as well as decrease in right ventricular ejection fraction, influence deterioration of left ventricular mechanics and systolic function in precapillary pulmonary hypertension. Left ventricular systolic function and left ventricular global longitudinal strain measurements using CMR-FT correlate with right ventricular dysfunction, and are associated with poor clinical outcomes in precapillary pulmonary hypertension patients.

## Figures and Tables

**Table 1:** Clinical characteristics of the PH patients.

Parameter	Non-Survival Group (*n* = 12)	Survival Group (*n* = 31)	*p* Value
Age (years)	56.0 [43.8–70.8]	55.0 [44.0–70.0]	0.840
Women/men (*n* (%))	7 (25)/5 (33.3)	21 (75)/10 (66.7)	0.561 *
IPAH (*n* (%))	3 (25)	10 (32.3)	0.643 *
PHSsc (*n* (%))	3 (25)	5 (16.1)	0.505 *
Eisenmenger syndrome (*n* (%))	4 (33.3)	9 (29.0)	0.784 *
CTEPH (*n* (%))	2 (16.7)	7 (22.6)	0.672 *
NYHA class 2/3/4 (*n* (%))	3 (25)/6 (50)/3 (25)	6 (19.4)/20 (64.5)/5 (16.1)	0.708 **
mPAP (mmHg)	56.5 [41.75–64.75]	54.0 [44.0–72.0]	0.753
6MWT (m)	270 [160.0–406.0]	313.5 [250.0–380.75]	0.488
NT-pro BNP (ng/mL)	2588.0 [1215.5–5521.0]	763.0 [238.0–2156.0]	0.022

Values are median [interquartile range] or *n* (%). NYHA—New York Heart Association functional class; 6MWT—six minutes walking test; NT pro BNP—brain natriuretic peptide; mPAP—mean pulmonary artery pressure. *p* value by Mann-Whitney U test * or Chi-square test **. NYHA *p* = 0.708, χ^2^ = 0.799, df = 2.

**Table 2:** Parameters of left and right ventricular function (CMR) and mechanics (FT) between the PH groups.

Parameter	Non-Survival Group (*n* = 12)	Survival Group (*n* = 31)	*p* Value
RVEDVI (mL/m^2^)	87.0 [76.0–112.3]	82.0 [68.0–101.0]	0.243
RVESVI (mL/m^2^)	63.5 [45.8–91.3]	48.0 [37.0–60.0]	0.060
RVEF (%)	37.5 [18.8–41.8]	42.0 [32.0–47.0]	0.088
LVEDVI (mL/m^2^)	60.5 [48.0–84.5]	62.0 [53.0–83.0]	0.862
LVESVI (mL/m^2^)	30.0 [20.75–45.25]	29.0 [18.0–38.0]	0.621
LVEF (%)	48.0 [38.8–60.5]	60.0 [50.0–66.0]	0.042
RV Free Wall LS (%)	−16.4 [−21.4–(−11.3)]	−18.1 [−22.9–(−13.8)]	0.243
RV Septum LS (%)	−9.95 [−13.85–(−6.3)]	−12.0 [−14.3–(−6.9)]	0.621
RV GLS (%)	−12.9 [−17.2–(−9.5)]	−14.1 [−16.9–(−11.0)]	0.399
LV GLS (%)	−12.4 [−19.0–(−7.8)]	−18.4 [−22.5–(−15.5)]	0.009
LV GCS (%)	−28.5 [−34.2–(−23.5)]	−32.0 [−36.8–(−28.2)]	0.221

Values are median [interquartile range]. RV—right ventricular; EDVI—end diastolic volume index; ESVI—end systolic volume index; EF—ejection fraction; LV—left ventricular; LS—longitudinal strain; GLS—global LS; GCS—global circumferential strain; *p* value by Mann-Whitney test.

**Table 3:** Risk of death based on univariable analysis using ROC test.

Parameter	Area under the ROC Curve, %	Sensitivity/Specificity, %	Survival/Non-Survival Group, *n* (%)	Non-Survival Group OR [95% CI]
LV GLS > −14.2%	76.1	77.8/93.5	2 (6.5)/8 (66.7)	20.3 [3.237–127.288]
RVESVI > 60 mL/m^2^	68.7	58.3/77.4	7 (22.6)/7 (58.3)	4.8 [1.156–19.925]
RVEF < 25.5%	66.9	41.7/90.3	3 (9.7)/5 (41.7)	6.667 [1.276–34.842]
LVEF < 52%	70.2	66.7/71.0	9 (29.0)/8 (66.7)	4.889 [1.171–20.408]

ROC—Receiver Operating Characteristics; LV—left ventricular; GLS—global longitudinal strain; RV—right ventricular; ESVI—end systolic volume index; EF—ejection fraction; OR—Odds Ratio; CI—Confidence Interval.

**Table 4:** Risk of death based on Cox regression analysis.

Parameter	Coefficient Beta	SE	*p* Value	HR [95% CI]
LV GLS > −14.2%	2.396	0.624	<0.001	10.974 [3.229–37.301]
RVESVI > 60 mL/m^2^	1.096	0.586	0.061	2.992 [0.949–9.433]
RVEF < 25.5%	1.253	0.587	0.033	3.501 [1.109–11.053]
LVEF < 52%	1.179	0.613	0.055	3.25 [0.977–10.809]
